# Supplementary material for: High-Performance Ammonia QCM Sensor Based on SnO2 Quantum Dots/Ti3C2Tx MXene Composites at Room Temperature
Source: Nanomaterials (Basel). 2024 Nov 16;14(22):1835. doi: 10.3390/nano14221835 (PMC11597869; doi:10.3390/nano14221835)
Supplement: Supplementary file 1 [file nanomaterials-14-01835-s001.zip › nanomaterials-3296190-supplementary.pdf]

Supplementary Information

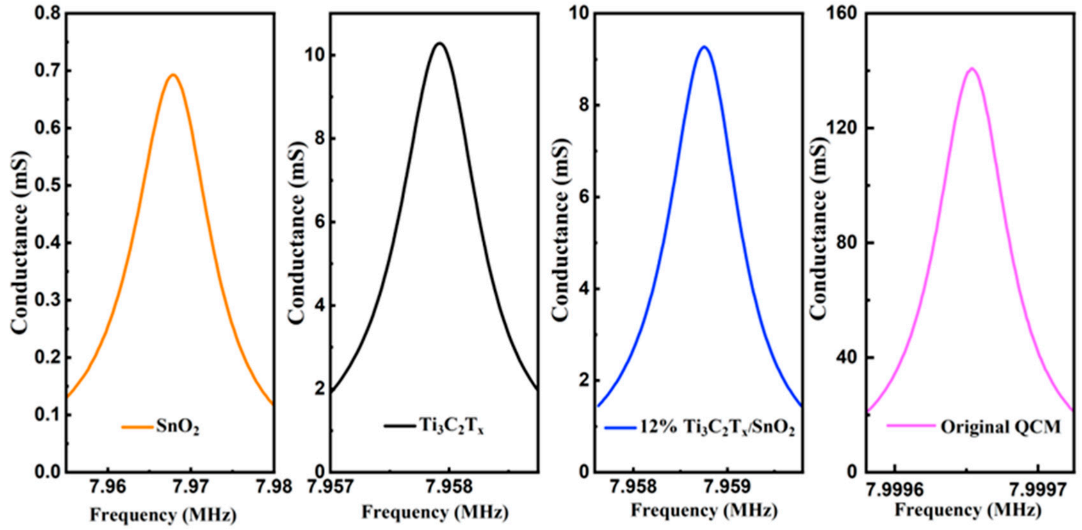

Figure S1. Conductivity of QCM sensors based on different materials.

As shown in Figure S1, following the deposition of the sensitive layer (SnO_2, $Ti_3C_2T_x$, $Ti_3C_2T_x/SnO_2$), a decrease in conductance was observed across all devices. Among them, SnO_2-based QCM exhibited the lowest conductance, which can be attributed to the long carbon chains of OLA and OA capping on SnO_2 inhibited carrier transport. The addition of $Ti_3C_2T_x$ in $Ti_3C_2T_x/SnO_2$ composites markedly enhanced the conductance of the composites, facilitating the formation of a robust ohmic contact between the composites and the electrodes and ensuring the vibration of the QCM sensor. In addition, an increase in the conductance of the complex increases the Q value of the sensor, which in turn increases the range of detection, according to previous reports [1].

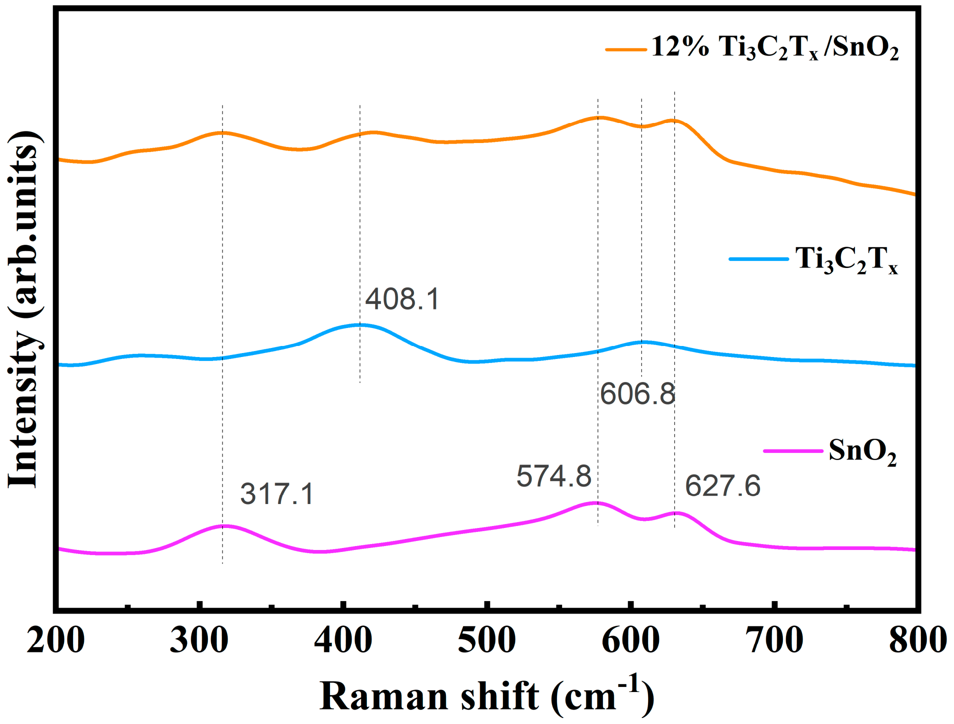

Figure S2. The Raman spectra of SnO_2, $Ti_3C_2T_x$ and 12% $Ti_3C_2T_x/SnO_2$ composites.

The Raman spectra of SnO_2, $Ti_3C_2T_x$ and 12% $Ti_3C_2T_x/SnO_2$ composites are shown in the Figure S2. SnO_2 exhibits three characteristic peaks at 317.1, 574.8 and 627.6 cm^{-1}. The $Ti_3C_2T_x$ MXene peaks are located at 408.1 and 606.8 cm^{-1} and correspond to the Eg vibrational modes of Ti with surface termination groups of -OH, -O and -F [2]. The characteristic peaks of the $Ti_3C_2T_x/SnO_2$ composites correspond to the peaks of SnO_2 and MXene, indicating that there were no vibrational changes during mixing and the composites were successfully prepared.

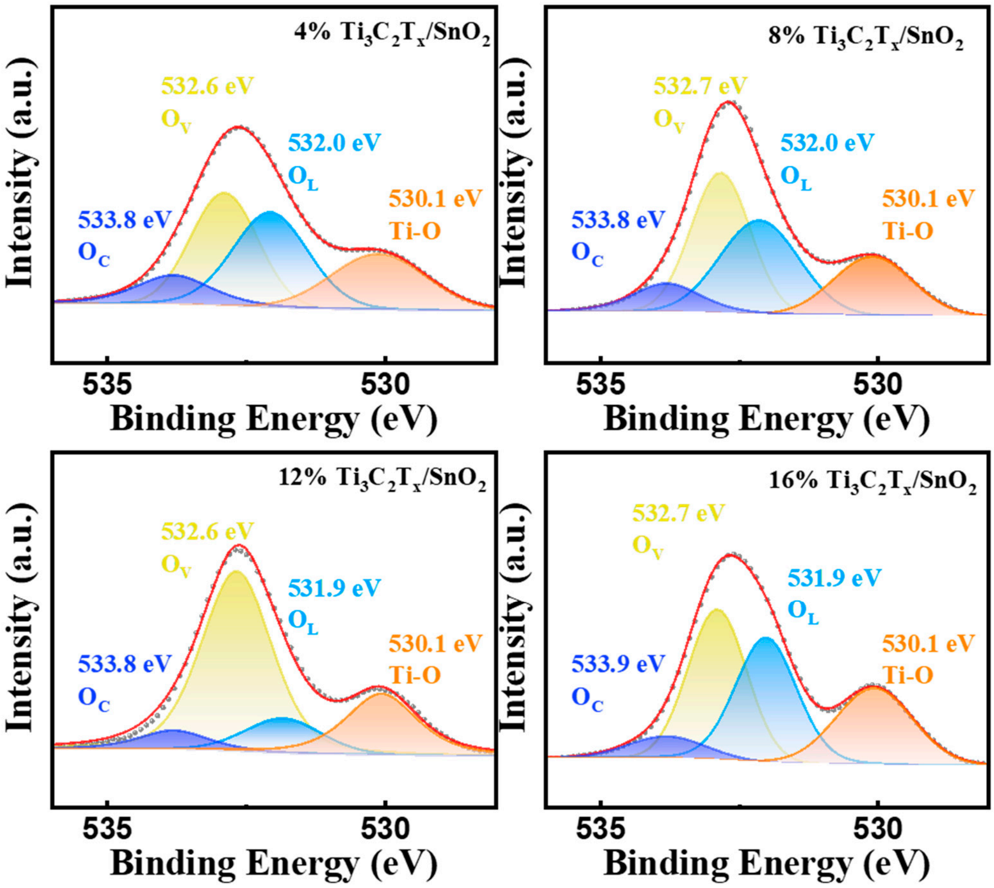

Figure S3. XPS spectra of O 1s of $Ti_3C_2T_x/SnO_2$ composites in different proportions.

The O1s results for the other $Ti_3C_2T_x/SnO_2$ samples are shown in Figure S3. Among them, the 12% $Ti_3C_2T_x/SnO_2$ had the highest O_V content of 58% (see Table S3). The presence of oxygen vacancies (O_V) can provide more sufficient active sites for gas adsorption, which is conducive to the adsorption of O_2 molecules and target gases.

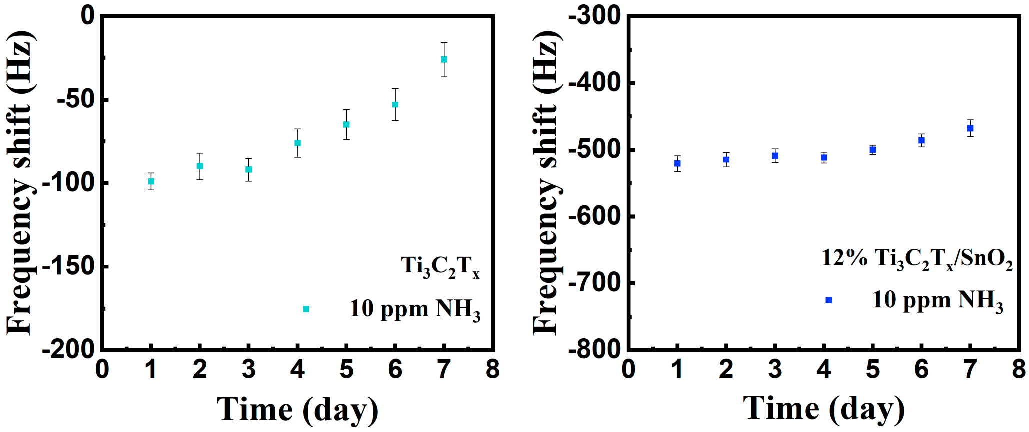

Figure S4. Long-term stability of the sensors based on $Ti_3C_2T_x$ and 12% $Ti_3C_2T_x/SnO_2$ composites at 80% RH.

In addition, the long-term stability of the sensor was evaluated in a high relative humidity (80% RH), as shown in Figure S3. Compared to pure $Ti_3C_2T_x$, the $Ti_3C_2T_x/SnO_2$ composites showed more stability, mainly due to the fact that the SnO_2 covering the surface of the $Ti_3C_2T_x$ blocked the oxygen gas direct contact with the $Ti_3C_2T_x$ to reduce the oxidation of $Ti_3C_2T_x$ MXene [3].

The QCM resonant frequencies were measured before and after coating the sensitive layer, and the mass of material deposited on the electrodes was calculated using the Sauerbrey equation [4], as shown in Eq. (S1).

$$\Delta f = \left(-2.26 \times 10^{-6} \frac{f_0^2}{A}\right) \Delta m \qquad \text{(S1)}$$

In the above equation, f_0 is the fundamental resonant frequency of the QCM, Δf is the frequency shift, and A is the surface area of the electrode. Table S1 provides a comprehensive illustration of the fundamental frequencies, frequency shifts and load masses of all the sensors.

Table S1. Preparation parameters of the fabricated sensors

QCM sensor	Fundamental frequency (Hz)	Frequency shift (Hz)	load mass (ng)
SnO_2	7999653	31914	43327
$Ti_3C_2T_x$ MXene	7999565	41658	56557
12 wt% $Ti_3C_2T_x$ MXene/ SnO_2	7999598	40847	55456

Table S2. Response values of all samples to different concentrations of ammonia gas.

NH_3 concentration	Response values of SnO_2 (Hz)	Response values of $Ti_3C_2T_x$ MXene (Hz)	Response values of 4 wt% $Ti_3C_2T_x/SnO_2$ (Hz)	Response values of 8 wt% $Ti_3C_2T_x/SnO_2$ (Hz)	Response values of 12 wt% $Ti_3C_2T_x/SnO_2$ (Hz)	Response values of 16 wt% $Ti_3C_2T_x/SnO_2$ (Hz)
0.2 ppm	/	/	/	/	−63±5.1	/

0.5 ppm	−26±6.1	/	−30±3.8	−51±5.3	−80±4.9	−40±5.3
1 ppm	−31±6.8	−15±2.3	−40±4.5	−74±5.9	−123±5.3	−61±5.8
2 ppm	−52±6.5	−26±3.5	−62±5.1	−120±6.7	−188±6.8	−94±6.7
4 ppm	−81±7.1	−43±5.4	−128±6.1	−232±4.6	−333±7.8	−166±4.9
6 ppm	−120±7.8	−56±6.2	−151±8.6	−303±8.6	−420±8.2	−220±8.6
8 ppm	−143±8.4	−80±6.8	−190±9.1	−365±7.9	−520±9.6	−270±7.5
10 ppm	−170±9.7	−90±7.9	−213±9.3	−426±8.9	−580±10.5	−310±8.9
20 ppm	−246±10.6	−119±9.1	−284±10.1	−569±11.3	−740±11.7	−419±9.3
30 ppm	−296±11.7	−143±8.9	−330±11.3	−640±12.1	−858±12.3	−529±10.3
40 ppm	−353±12.3	−156±9.3	−384±12.1	−769±11.5	−1000±16.4	−620±11.6
50 ppm	−399±13.1	−198±11.2	−423±16.7	−846±15.8	−1100±18.3	−690±13.5

Table S3. O1s XPS peak areas for all $Ti_3C_2T_x/SnO_2$ composites.

QCM sensors	Ti-O peak area	O_L peak area	O_V peak area	O_C peak area
4% $Ti_3C_2T_x/SnO_2$	23%	33%	32%	12%
8% $Ti_3C_2T_x/SnO_2$	21%	33%	37%	10%
12% $Ti_3C_2T_x/SnO_2$	23%	12%	58%	7%
16% $Ti_3C_2T_x/SnO_2$	25%	33%	36%	7%

References

1. Liu, P.; Ma, X.; Feng, L.; Chen, Y.; Lu, J.; Zhang, L.; Pei, Z., High-Quality-Factor Quartz Crystal Microbalance Ammonia Sensor Based on Self-Assembled Film. *Journal of Electronic Materials* **2022,** 52, (2), 1314-1322. http://dx.doi.org/10.1007/s11664-022-10018-w
2. Zhang, B.; Li, Z.; Li, C.; Li, M.; Fu, C.; Tao, R.; Zha, X.-h.; Li, H.; Luo, J., High-sensitive ppb-level ammonia QCM sensor based on sulfur doped Ti3C2Tx MXene. *Sensors and Actuators A: Physical* **2023,** 350. http://dx.doi.org/10.1016/j.sna.2022.114138
3. Zhang, H.-F.; Xuan, J.-Y.; Zhang, Q.; Sun, M.-L.; Jia, F.-C.; Wang, X.-M.; Yin, G.-C.; Lu, S.-Y., Strategies and challenges for enhancing performance of MXene-based gas sensors: a

review. *Rare Metals* **2022**. http://dx.doi.org/10.1007/s12598-022-02087-x

4. Kan, H.; Li, M.; Li, H.; Li, C.; Zhou, J.; Fu, C.; Luo, J.; Fu, Y., A novel quartz-crystal microbalance humidity sensor based on solution-processible indium oxide quantum dots. *RSC Adv* **2019,** 9, (66), 38531-38537. http://dx.doi.org/10.1039/c9ra06385d
